# Is the tail of the pancreas always tumor-infiltrated when macroscopically affected during cytoreductive surgery? A clinicopathological study and experience from a high-volume center

**DOI:** 10.1186/s12957-025-03954-4

**Published:** 2025-07-24

**Authors:** Miklos Acs, Jozef Zustin, Niklas Bogovic, Pompiliu Piso, Sebastian Blaj

**Affiliations:** 1 Department of Surgery, University Medical Center Regensburg, D-93053 Regensburg, Germany; 2 Gerhard Domagk Institute of Pathology, University Medical Center Münster, D-48149 Münster, Germany; 3 Institute of Pathology, University of Regensburg, D-93053 Regensburg, Germany; 4 Department of General and Visceral Surgery, Hospital Barmherzige Brüder, D-93049 Regensburg, Germany

**Keywords:** Cytoreductive surgery, Hyperthermic intraperitoneal chemotherapy, Pancreatic tail resection, Peritoneal surface malignancy, Pancreatic fistula

## Abstract

**Background:**

Distal pancreatic resection during cytoreductive surgery (CRS) and hyperthermic intraperitoneal chemotherapy (HIPEC) is rare, with limited knowledge available. Therefore, a retrospective observational study was conducted using the data registry of a single institution to identify patients that underwent distal pancreatic resection during CRS + HIPEC.

**Methods:**

All resected pancreatic specimens were examined for invasive parenchymal tumor infiltration. Pre-, peri-, and postoperative variables and their associations were analyzed.

**Results:**

Over a period of more than a decade, 31 of 1275 patients (2.43%) underwent distal pancreatic resection as part of CRS. Infiltration of the pancreatic parenchyma was confirmed in almost one-third (29.03%) of the cases. Postoperative pancreatic fistulas occurred in 25.81% of patients (87.5% Grade B; 12.5% Grade C). The need for distal pancreatic resection was closely related to tumor burden in the left upper abdomen, with 87% of patients requiring peritonectomy of the left upper abdomen in addition to visceral resection. Pancreatic infiltration (*n* = 9/31) was diagnosed in 3 cases of gastric carcinoma, 2 cases of colorectal carcinoma, 2 cases of primary peritoneal carcinoma, 1 case of ovarian carcinoma, and 1 case of mucinous appendiceal carcinoma. Postoperative pancreatic fistulas were more frequently associated with primary tumors of the large intestine (87.50% vs. 30.43%; *P* = 0.0094), and a tendentiously longer total hospital stay was required for the “with pancreatic fistula” group (32.50 ± 19.93 days vs. 21.78 ± 10.14 days), with no impact on patient survival.

**Conclusions:**

Accepting a slightly increased morbidity, distal pancreatic resection is a reasonable approach to achieve complete macroscopic tumor resection. Nonetheless, our study shows that apparent tumor invasion is histologically rare in cases with favorable tumor biology, such as low-grade pseudomyxoma peritonei. Therefore, pancreatic resection should be avoided in cases of mucinous tumors to prevent fistula formation.

**Supplementary Information:**

The online version contains supplementary material available at 10.1186/s12957-025-03954-4.

## Background

Multimodal treatment of patients with peritoneal metastases—including cytoreductive surgery (CRS) and hyperthermic intraperitoneal chemotherapy (HIPEC)—has been practiced for approximately three decades and has significantly improved oncologic outcomes and prognoses in selected patients. The primary goal of CRS is to surgically remove all visible peritoneal metastatic lesions in the abdominal cavity, while HIPEC targets free cancer cells and microscopic peritoneal metastases that may remain after surgery. Achieving macroscopic tumor clearance typically requires parietal peritoneal stripping and multivisceral resection.In patients with extensive disease near the splenic hilum, complete macroscopic resection may not be feasible without sacrificing the pancreatic tail. In such cases, splenectomy—with or without distal pancreatectomy—may become necessary. Indications for distal pancreatectomy during CRS include tumor involvement of the splenic hilum, direct infiltration of the pancreatic capsule or parenchyma, or iatrogenic pancreatic injury. At high-volume centers, the need for distal pancreatectomy in the context of CRS has been reported in 2.5–6.2% of cases. However, this procedure is associated with significant morbidity (ranging from 12 to 74%, partly due to postoperative pancreatic fistulas) and a reported mortality rate of 0.5–6%.Our understanding remains limited regarding how tumors of various origins and biological behaviors infiltrate surrounding organs—particularly the pancreas—when they are macroscopically affected. Therefore, the primary aim of this study was to evaluate the pathological findings of resected pancreatic specimens for tumor infiltration across different tumor types. A secondary aim was to describe our institutional experience and perioperative outcomes in patients undergoing CRS and HIPEC in the context of distal pancreatic involvement.

Multimodal treatment of patients with peritoneal metastases, including cytoreductive surgery (CRS) and hyperthermic intraperitoneal chemotherapy (HIPEC), has been described for about three decades and has substantially improved the oncological prospects and prognosis of selected patients. The ultimate aim of CRS is to surgically remove all visible peritoneal metastatic lesions in the abdominal cavity, while HIPEC targets the remaining free cancer cells and microscopic metastases to the peritoneum [1]. For surgical macroscopic tumor clearance, parietal peritoneal stripping and multivisceral resection are required. In patients with extensive disease in the splenic hilum, where satisfactory separation of the pancreatic tail is not feasible to achieve complete macroscopic tumor resection, a splenectomy—with or without removal of the distal pancreas—may be necessary [2]. Distal pancreatic resection during CRS is not just indicated in the cases of tumor involvement of the splenic hilum, pancreatic capsule, or the pancreas, but also may be necessary due to iatrogenic injury of the pancreas [3]. The necessity for distal pancreatic resection to gain complete macroscopic tumor resection in high-volume centers ranges between 2.5–6.2% [4–7]. This procedure, as part of CRS, is associated with a significant morbidity ranging between 12 and 74% (partly due to postoperative pancreatic fistulas) and with a mortality rate of 0.5–6% [8–10]. However, our understanding of how tumors of different origins, biology, and aggressiveness can invasively infiltrate the tumor-affected organs, and thus currently the pancreas, is limited. Therefore, the primary aim of this study was to report the pathological results of the resected pancreas specimens for possible tumor infiltration from different tumor origins, and the secondary aim was to describe our experience and perioperative outcomes of patients with peritoneal surface malignancies involving the distal pancreas.

## Results

Between January 2011 and December 2024, a total of 1275 CRS + HIPEC procedures were performed in a German tertiary referral center for peritoneal surface malignancies. Of these, 31 cases (2.43%) required distal pancreatic resection. The median age of the patients was 57 years (ranging from 24 to 77 years of age), and more than half of them were females (54.87%). The primary origin of the cancer was the appendix in 9 cases (29%) [mucinous adenocarcinoma: 1 (3.2%); low-grade appendiceal mucinous neoplasm (LAMN): 8 (25.8%)], the colorectum in 5 cases (16.1%) (colon: 3 (9.7%); rectum: 2 (6.4%)), the stomach in 7 cases (22.6%), mesothelioma in 2 cases (6.45%), the ovaries in 3 cases (9.68%), and the peritoneum in 5 cases (16.1%) [high-grade serous: 3 (9.7%); desmoplastic small round cell tumors: 1 (3.2%); unspecified: 1 (3.2%)]. The complete list of pre-, peri-, and postoperative parameters of the study population can be read in Tables S1.

### Which clinicopathological parameters are associated with tumor infiltration and/or fistula of the pancreas?

To investigate whether any of the clinicopathological parameters are more frequent in those patients who either had invasive parenchymal tumor infiltration of the pancreas or developed postoperative pancreatic fistula, the study population was further subdivided into subgroups. 9 (29.03%) and 8 (25.81%) patients showed invasive patterns or developed postoperative pancreatic fistulas, respectively. The resection technique of the pancreatic tail was also considered as a potential parameter to be investigated; however, no difference could be found between the subgroups (open method ((with scalpel)): 19.35%, endo-GIA: 58.06%, TA 60: 22.58%) in any of the comparisons.

**Fig. 1:**
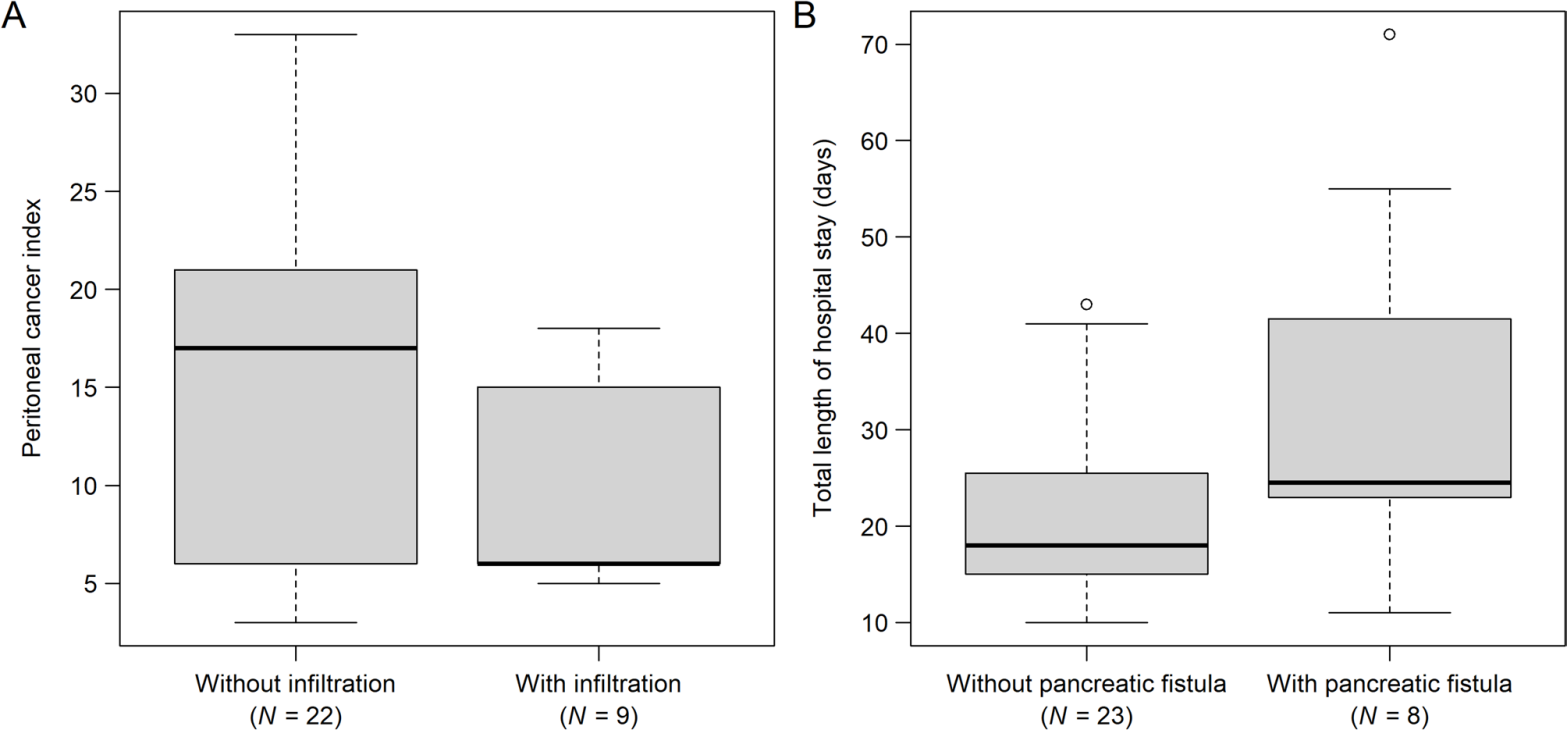
Lower peritoneal cancer index values were found in those patients without invasive parenchymal tumor infiltration of the pancreas (crude *P* = 0.0322; **A**), and tendentiously longer total hospital stay was observed in the case of those patients where postoperative pancreatic fistula developed (crude *P* = 0.0969; **B**)

When comparing the patients based on the presence of pancreatic tumor infiltration, only 1 of the 9 appendiceal cancers was affected by tumorous infiltration. This was the case for the only mucinous adenocarcinoma, while none of the LAMN cases were affected. It was found that the peritoneal cancer index (PCI) of patients with invasive parenchymal tumor infiltration of the pancreas was tendentially lower compared to those without infiltration (crude *P* = 0.0322; adjusted *P* = 0.4186 Fig. 1A). Peritonectomy of the omental bursa (with infiltration: 11.11%; without infiltration: 68.18%; crude *P* = 0.0033), colon–rectum anastomosis (with infiltration: 0%; without infiltration: 31.82%; crude *P* = 0.0766), and rectosigmoid resection (with infiltration: 0%; without infiltration: 36.36%; crude *P* = 0.0705) were less frequent. In contrast, small bowel resection (with infiltration: 55.56%; without infiltration: 13.64%; crude *P* = 0.0159) and cholecystectomy (with infiltration: 100%; without infiltration: 59.09%; crude *P* = 0.0315) were more frequent in the “with tumor infiltration” group. Among postoperative complications, pleural effusion occurred more frequently in patients in the “with tumor infiltration” group (with infiltration: 44.44%; without infiltration: 9.09%; crude *P* = 0.0434).

Additionally, 22.6% of the patients did not undergo peritonectomy in the left upper quadrant. A review of their medical histories revealed that all patients had been treated for recurrent tumor disease and had previously undergone deperitonealization of the left upper quadrant during their initial surgery. The comparisons between with and without pancreatic tail infiltration are shown in Table S1 where after correction for multiple testing, all adjusted p values were insignificant.

Pancreatic fistulas were observed in 25.81% of the patients undergoing a distal pancreatic resection (*n* = 7/8; 87.5% Grade B and *n* = 1/8; 12.5% Grade C fistula). The need for distal pancreatic resection was shown to be closely related to the tumor burden in the left upper abdomen, with 87% of patients requiring peritonectomy of the left upper abdomen in addition to visceral resection. Pancreatic infiltration (*n* = 9/31) was diagnosed in 3 (33.3%) gastric carcinoma, 2 (22.2%) colorectal carcinoma, 2 (22.2%) primary peritoneal carcinoma, 1 (11.1%) ovarian carcinoma, and 1 (11.1%) mucinous appendiceal carcinoma patients.

The mortality rate after pancreatic tail resection or, in the case of a pancreatic fistula, was nil. Two patients were reoperated due to pancreatic fistula and consecutive intra-abdominal complications.

Pancreatic fistulas were highly associated with tumors originating from the large intestine (appendix or colorectum), compared to other tumor sites (87.50% vs. 30.43%; crude *P* = 0.0094). Stomach resection (with fistula: 12.50%; without fistula: 65.22%; crude *P* = 0.0121) and stomach–small bowel anastomosis (with fistula: 12.50%; without fistula: 56.52%; crude *P* = 0.0454) were needed less frequently in patients with pancreatic fistulas. As pancreatic fistulas are major complications following surgery, as an expected result, lower Clavien-Dindo grades never occurred in the “with pancreatic fistula” subgroup (0% vs. 56.52%; crude *P* = 0.0156). Furthermore, consistent with the previous findings, the overall length of hospital stay was tendentially longer in the pancreatic fistula subgroup (without fistula: 21.78 ± 10.14 days; with fistula: 32.50 ± 19.93 days; crude *P* = 0.0969; Fig. 1B). The complete list of comparisons between patients with and without pancreatic fistula can be found in Table S1.

### What is the impact of pancreatic infiltration and/or fistula or resection technique on patient survival?

Follow-up data were also collected for the study population to calculate overall survival (OS). It should be highlighted, however, that for 8 out of 31 patients, no further follow-up data could be obtained after hospital discharge. For this reason, survival results could only be calculated from the data of 23 patients in total, meaning that in some cases, these results may only be interpreted as trends. Both univariate and multivariate survival models were created. To reduce bias due to heterogeneity and to obtain valid results for the subgroups, the baseline hazard was adjusted for the location of the primary tumors in all survival models.

**Fig. 2:**
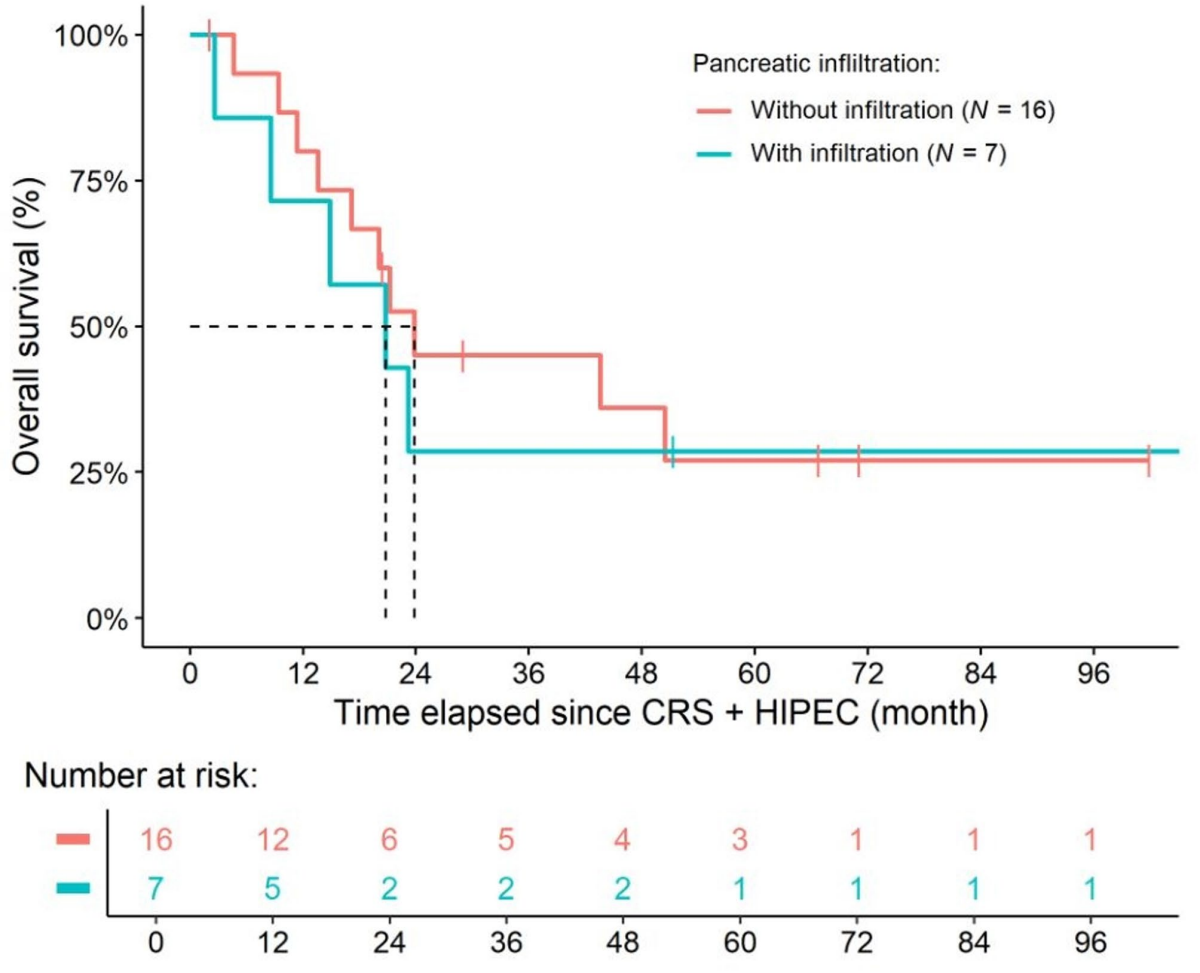
Overall survival of the study population grouped on the presence of invasive parenchymal tumor infiltration. In the comparison, no difference could be justified between the two subgroups

During the observational period, 15 of the 23 patients (65.22%) died, with a median OS of 21.22 months [95% confidence interval (95% CI): 17.15– not reached]. There was no significant difference in OS between patients with and without invasive parenchymal tumor infiltration (*P* = 0.5020, Fig. 2A). Similarly, postoperative pancreatic fistulas did not affect the OS of patients (*P* = 0.2660).

The effect of the investigated parameters was further assessed in a multivariate setting through the creation of two multivariate survival models. First, the combined effect of the three main parameters of interest—tumor infiltration, pancreatic fistula, and resection technique—was evaluated. This was followed by a second model that included additional clinicopathological parameters. In the latter model, tumor infiltration, pancreatic fistula, age, sex, PCI, and Sugarbaker’s completeness of cytoreduction score (CC-score) were analyzed.

It was found that in the first model (Table 1), only the use of the endo-GIA technique was associated with longer patient survivals (*P* = 0.0499), while none of the other parameters showed any effect on OS. Similarly, the second model did not show that any of the parameters had a particularly significant effect on patient survival (Table 2).

**Table 1:** Multivariate survival model investigating the combined effect of the study’s three main parameters of interest on the overall survival of the study cohort

Parameter	HR	95% CI	*P* value
Invasive parenchymal tumor infiltration of the pancreas [No (ref.) vs. Yes]	2.6730	0.5406–13.2158	0.2279
Postoperative pancreatic fistula [No (ref.) vs. Yes]	0.4888	0.0389–6.1402	0.5793
Distal pancreatectomy resection technique:			
- Open (ref.) vs. endo-GIA	0.1350	0.0182–0.9994	0.0499
- Open (ref.) vs. TA 60	0.6378	0.0733–5.5515	0.6838
- endo-GIA (ref.) vs. TA-60	4.7260	0.4995–44.7200	0.1756

CI: confidence interval; endo-GIA: endo gastrointestinal anastomosis stapling device; HR: hazard rate; ref.: reference category; TA 60: thoracoabdominal stapling device

**Table 2:** Multivariate survival model investigating the combined effect of the study’s two main parameters of interest and additional clinicopathological parameters on the overall survival of the study cohort

Parameter	HR	95% CI	*P* value
Invasive parenchymal tumor infiltration of the pancreas [No (ref.) vs. Yes]	4.3133	0.2329–79.8900	0.3260
Postoperative pancreatic fistula [No (ref.) vs. Yes]	0.3541	0.0116–10.8450	0.5520
Age (year)	1.0080	0.9516–1.0680	0.7870
Sex [Male (ref.) vs. Female]	5.4022	0.1117–261.1860	0.3940
PCI (unit)	1.0977	0.9032–1.3340	0.3490
CC-score [CC-0 (ref.) vs. CC-1]	0.0450	0.0005–4.1220	0.1780

CC-score: Sugarbaker’s completeness of cytoreduction score; CI: confidence interval; HR: hazard rate; PCI: peritoneal cancer index at exploration; ref.: reference category

## Discussion

The tumor-free margin of the pancreas is of paramount importance for confirming macroscopic complete cytoreduction (CC-0) when a patient undergoes distal pancreatic resection as part of cytoreductive surgery. Therefore, both overestimation and underestimation of tumor extent during surgery are possible [11]. This discrepancy between histopathological tumor detection and morphological tumor involvement was highlighted convincingly by Bhatt et al. [11], who advocated for the use of more frequent frozen sections during CRS. Distal pancreatic resection during CRS differs substantially from the same procedure performed for pancreatic cancer. Distal pancreatic resection during CRS differs substantially from the same procedure for pancreatic cancer. In the case of CRS, there is no need for radical, oncological resection of the pancreas; therefore, a more limited resection to the left may be performed. The macroscopic tumor involvement of the pancreatic tail does not necessarily mean the tumorous infiltration of the parenchyma, which is the main finding of our current study. To our knowledge, this is the first description of this subject in the context of peritoneal surface malignancies. Notably, 22 out of 31 patients (70.97%) showed no invasive infiltration of the pancreatic tissue despite intraoperatively apparent morphological tumor seeding. In line with advances in our understanding of the biology of peritoneal metastatic disease, more accurate prediction of neoplastic aggressiveness and behavior is becoming possible—not only at the systemic level but also within individual organs involved. Hence, in cases of aggressive tumor biology—such as gastric carcinoma, high-grade serous ovarian or primary peritoneal carcinoma, mucinous adenocarcinoma of the appendix, and mesothelioma—particular attention and repeated frozen sections are required to ensure tumor-free resection of the pancreatic margin. An interesting observation was that none of the LAMN cases was affected by the parenchymal infiltration. Based on this, we hypothesize that invasive tumor growth into the pancreatic parenchyma is less likely to be expected in this type of tumor; however, further thorough research on this subject is needed. Furthermore, since 2016, we have adopted a more restrictive policy regarding distal pancreatic resection, particularly for low-grade pseudomyxoma peritonei. This change was prompted by the relatively high number of pancreatic fistulas observed during the initial period (from 2011 to the end of 2015). Retrospectively, 6 pancreatic fistulas occurred during 476 CRS + HIPEC procedures and 19 distal pancreatic resections performed during that period (1.2% and 31.5%, respectively). In contrast, from 2016 to the end of 2024, only 2 pancreatic fistulas were observed among 799 CRS + HIPEC procedures and 12 distal pancreatic resections (0.25% and 16.7%, respectively), representing a significantly lower event rate (*p* < 0.05).

Grade 3 and 4 complications are common after pancreatic tail resection as a part of multivisceral surgery, occurring up to 40% [4, 12]. The main surgical complications of pancreatic leakage are intra-abdominal abscess and postoperative bleeding, as well as new onset pancreatogenic diabetes mellitus (also known as type 3c diabetes [13]) with an incidence of 7.5% after distal pancreatectomy [14, 15]. Similarly, the pancreatic fistula rate after distal pancreatectomy ranges from 3–26% [3, 10, 16, 17]. A large international multicentric study by Schwarz et al. [2] included 118 patients with peritoneal surface malignancy undergoing distal pancreatic resection as a part of CRS and HIPEC. The 90-day postoperative mortality was 7.6%, and the rate of pancreatic fistula was 33%. In our current study, there was no perioperative mortality, and the rate of pancreatic fistula was 25.81%. The reoperation rate of the complete study cohort was 25.81%, which is similar to other previous substantial reports [2, 4]. Furthermore, Down-Canner et al. [18] showed in their comparative study that postoperative pancreatic fistulas are more severe when distal pancreatectomy is combined with CRS and HIPEC, compared to pancreatectomy alone. Both the studies by Kusamura et al. [16] and Downs-Canner et al. [18] hypothesized that the use of HIPEC may predispose to more severe pancreatic fistulas due to the cytotoxic effects of HIPEC and potentially impaired wound healing. Nevertheless, the study by Down-Canner et al. [18] compared patients undergoing distal pancreatectomy during CRS and HIPEC for peritoneal surface malignancy with those patients undergoing minimally invasive or open distal pancreatectomy without HIPEC for locally resectable pancreatic adenocarcinomas. This clearly limits the significance of the study’s conclusions, as two entirely different patient populations were compared. A more appropriate comparison would involve patients undergoing distal pancreatic resection in the context of CRS, with and without HIPEC.

The issue of pancreatic fistulas warrants further discussion. At our center, during HIPEC procedures, we routinely place five 24 Ch closed drains, one of which is positioned in the former splenic lodge following combined splenectomy and distal pancreatic resection. The rationale for placing a drain after pancreatic tail resection is to detect potential pancreatic leaks, which can lead to abdominal abscesses or pancreatic-cutaneous fistulas. Nevertheless, a multicenter randomized trial reported no significant difference in the incidence of pancreatic fistulas with or without intraperitoneal drainage (18% vs. 12%, respectively) [19]. Not having a drain was associated with a higher incidence of intra-abdominal fluid collection (9% vs. 22%) [19], which aligns with our experience: early drain removal was associated with the need for placement of a new drainage system, guided by sonography or CT scan, in nine patients (29.03%, data not presented). On the other hand, a recent multicenter study reported that omitting drain placement after distal pancreatectomy was associated with a lower rate of major complications (Clavien–Dindo ≥ 3), including pancreatic fistulas [20]. It should be noted, however, that these findings are based not only on peritoneal surface malignancies but also on other conditions that may necessitate distal pancreatectomy. A novel result of our study was the strong association between postoperative pancreatic fistulas and primary tumors originating from the large intestine. To our knowledge, this association has not been previously reported. However, the number of cases supporting this observation was very limited, and thus we can only speculate on the underlying cause. Multicenter studies and/or larger cohorts are needed to confirm this association in future populations.

According to most experts in the field of pancreatic surgery, there is no difference in the fistula rate after pancreatic tail resection between handsewn and stapler techniques [21]. imilarly, a Cochrane review from 2015 found no significant differences in pancreatic fistula rates, overall postoperative mortality, or operative time between stapler resection and scalpel resection followed by hand-sewn closure of the pancreatic remnant [22]. In alignment with these authors, we also did not find any superiority among the techniques used, except for a slight tendency toward longer OS in patients operated on with the endo-GIA technique—an effect that was more pronounced in the multivariate analysis. As highlighted by a recent multicentric retrospective analysis of 2026 patients, the occurrence of pancreatic fistula after distal pancreatic resection may depend more on patient-related factors than on the surgical technique itself [23].

The management of postoperative pancreatic leakage in our study was similar to that described in the literature, both in the context of distal pancreatectomy for pancreatic neoplasms and after CRS and HIPEC. Accordingly, external drainage was the treatment of choice, typically accompanied by the need for antibiotic therapy [18, 24]. Another promising treatment option—successfully used in one patient—was the placement of a lumen-apposing metal stent through the stomach into the intra-abdominal pancreatic fluid collection. The continued development of this endoscopic approach, particularly Hot AXIOS stent placement, is increasingly being applied for the treatment of pancreatic fluid collections via the gastrointestinal tract [25].

### Limitations of the study

The present retrospective study has several limitations. The main limitations are (1) its retrospective design and the resulting potentially inevitable bias, (2) the low sample size, (3) the data presented here originated from a single center, and (4) the high heterogeneity of the patient population. Further limitations include the limited data on survival. Moreover, we did not detect long-term impairment of exocrine pancreatic function in terms of newly onset type 3c diabetes, even in cases where an extreme length of hospitalization was required following CRS + HIPEC.

## Conclusion

For the sake of achieving complete macroscopic tumor resection in CRS, distal pancreatectomy is a reasonable procedure, accepting increased morbidity. Nevertheless, following distal pancreatectomy, microscopic histological examination is necessary to rule out an R1 parenchymal resection. According to our findings, histopathologically confirmed tumor invasion of the pancreatic parenchyma was observed just in a few cases, and the development of postoperative pancreatic fistulas appeared to be strongly associated with primary tumors originating from the large intestine. Therefore, careful consideration is needed when indicating distal pancreatic resection. If resection is performed, a frozen section is recommended to exclude microscopically incomplete (R1) resection of the pancreatic parenchyma. Apparent tumor invasion was also observed in a few cases with favorable tumor biology—such as low-grade pseudomyxoma peritonei—where the potential for true invasion was minimal. As such, pancreatic resection should be avoided in cases of LAMN to prevent fistula formation, given the very low probability of histological infiltration of the pancreatic tail.

## Methods

### Ethics approval

All the study participants agreed to data recording for the national HIPEC registry of the German Society for General and Visceral Surgery (DGAV) and to the use of their anonymized data for quality assurance and research purposes by written and verbal informed consent prior to surgery. Ethical review and approval were waived for this study due to the its retrospective nature. The study was conducted in accordance with the guidelines of the Declaration of Helsinki.

### Patients and study design

A retrospective observational study was conducted. Data from a total of 1275 patients who attended the Department of General and Visceral Surgery at Hospital Barmherzige Brüder, Regensburg, Germany, between January 2011 and December 2024, were assessed. Among them, 31 consecutive patients who underwent pancreatic tail resection within the framework of CRS + HIPEC were identified. All patients were treated based on multidisciplinary recommendations. Patients who required pancreatic tail resection due to iatrogenic injury were excluded from the study.

### Clinicopathological and CRS + HIPEC details

Clinicopathological data were obtained from prospectively collected databases and electronic medical records. The extent of peritoneal dissemination was assessed preoperatively using abdominal and chest CT scans. Patients with questionable operability due to extensive tumor involvement on preoperative imaging underwent diagnostic laparoscopy prior to surgery.

The indication for distal pancreatectomy was extensive tumor infiltration at the splenic hilum with macroscopic involvement of the pancreatic tail. In cases following splenectomy, the indication was tumor infiltration of the left upper abdomen involving the pancreatic tail. In some instances, preoperative CT scans already suggested suspected infiltration. Nevertheless, the decision to resect the pancreatic tail was made intraoperatively on an individual basis for each patient.

During CRS, the completeness of cytoreduction (CC) was scored as proposed by Sugarbaker [26]: CC-0: no residual disease; CC-1: residual nodules measuring less than 2.5 mm; CC-2: residual nodules measuring between 2.5 and 2.5 cm; and CC-3: residual nodules greater than 2.5 cm. The extent of peritoneal disease was assessed by using the Peritoneal Cancer Index (PCI), which ranges from 1 to 39 [27]. Immediately following CRS, closed HIPEC with a goal temperature of 42 °C with intraperitoneal chemotherapy was administered for 30, 60, or 90 min.

Postoperative adverse events were categorized according to the Clavien-Dindo classification, and major complications were defined as Grade ≥ 3 [28]. OS was calculated from the date of surgery (CRS + HIPEC) to the date of death from any cause. The follow-up of patients was terminated on 30 September 2022, and the patients alive at this time point were right-censored. The pancreatic fistulas were classified and treated according to the International Study Group for Pancreatic Fistula (ISGPF) recommendations in grades A-C [29, 30].

### Histopathological processing of pancreas tail specimens

**Fig. 3:**
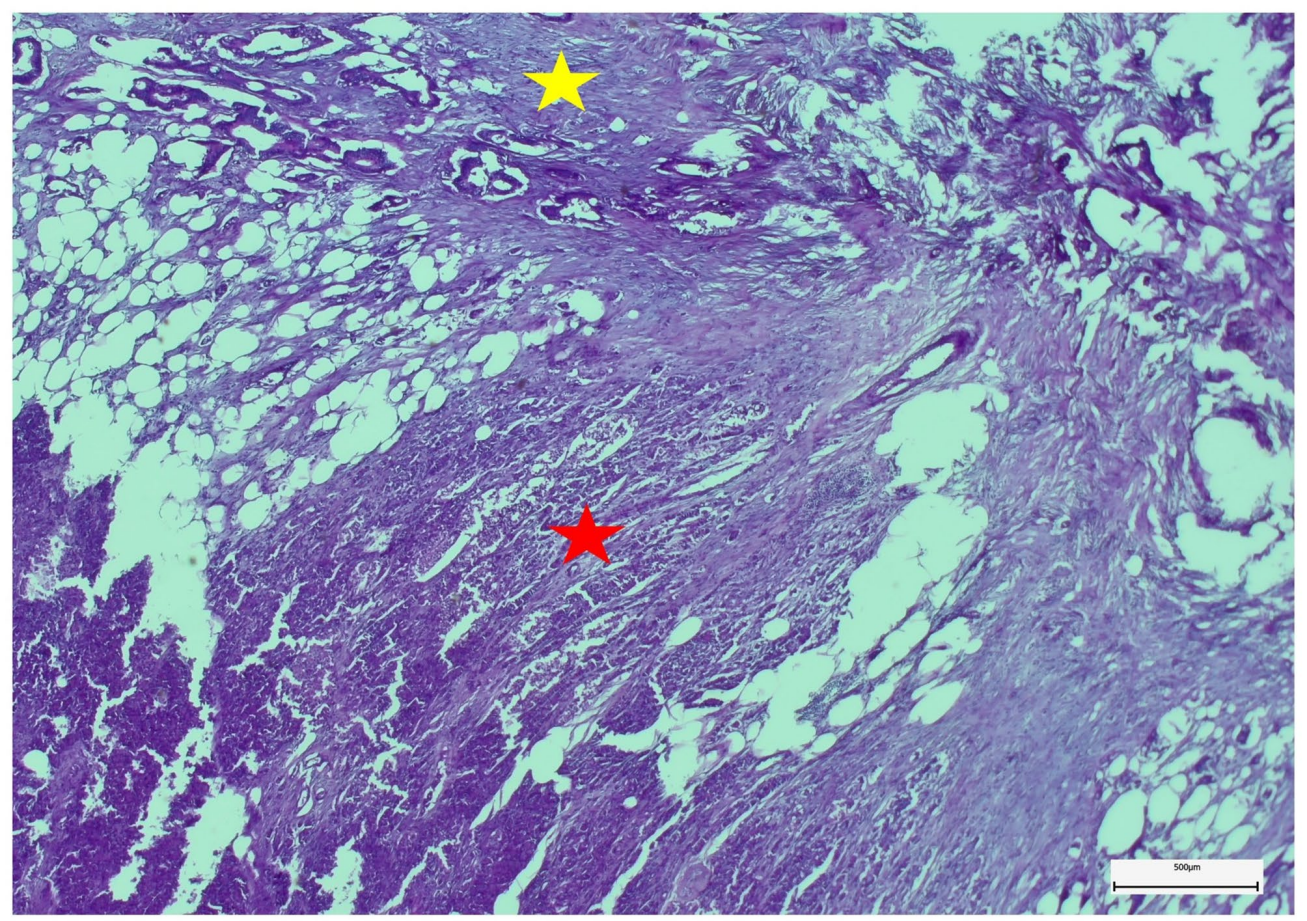
Microscopically, atypical ductal neoplastic proliferates infiltrate both peripancreatic fibro-lipomatous (yellow star) and pancreatic tissues (red star). The neoplastic invasive growth is associated with fibrosis and regressive changes of soft tissues (embedding in paraffin wax, staining method: hematoxylin-eosin, original magnification: ×4)

All pancreas tail specimens were examined histopathologically in detail, especially for the tumorous infiltration of the pancreatic parenchyma (Fig. 3).

### Description of the distal pancreatectomy procedure

In cases of distal pancreatectomy, the pancreas was transected to the right of the pathological area, representing a more limited resection than classical distal pancreatectomy, in which the transection line may extend as far as the neck of the gland.

Over the years, three methods have been used for pancreatic tail resection: (1) In the open method, the splenic artery and vein are first suture ligated, then the pancreas is divided with a scalpel, and the pancreatic duct is sutured over with a 5.0 Prolene suture. The pancreatic resection area is then sutured over with a 4.0 Prolene suture using a continuous technique in a “fish-mouth” style. (2) In the “TA 60” method, the tail of the pancreas is divided with a thoracoabdominal stapling device (TA 60), and the pancreatic stump is sutured over with single stitches using Monosyn or Maxon of thickness 5.0. And 3.), an endo gastrointestinal anastomosis stapling device (endo GIA) with Goretex mesh staple reinforcement is used for stapling the tail of the pancreas.

### Statistical analysis

Statistical analyses were performed using R for Windows, version 4.4.3 (R Foundation for Statistical Computing, 2025, Vienna, Austria). To minimize bias resulting from the low sample size, robust stochastic tests were applied, including the Brunner–Munzel test [31] and the Fisher’s exact test, for group comparisons. OS data were analyzed using extended Cox proportional hazards models with baseline hazard correction to account for data heterogeneity (R packages *survival*, version 3.8-3, and *survminer*, version 0.5.0). Parameter selection for multivariate survival models was based not on univariate *P* values but on relevant literature and the medical/clinical importance of the given parameter. A *P* value < 0.05 was considered statistically significant, and *P* values were corrected with the Bejamini–Hochberg method [32] to address the multiple-comparisons problem. Continuous, survival, and count data were expressed as the mean ± standard deviation, the HR with a 95% CI, and the number of observations (percentage), respectively.

## Electronic supplementary material

Below is the link to the electronic supplementary material.

Supplementary Material 1

## Data Availability

No datasets were generated or analysed during the current study.

## Abbreviations

CC: Completeness of cytoreduction
CC-0: Complete cytoreduction
CC-1: Residual nodules measuring less than 2.5 mm
CC-2: Residual nodules measuring between 2.5 and 2.5 cm
CC-3: Residual nodules greater than 2.5 cm
CI: Confidence interval
CRS: Cytoreductive surgery
DGAV: German Society for General and Visceral Surgery
endo GIA: Endo gastrointestinal anastomosis stapling device
HIPEC: Hyperthermic intraperitoneal chemotherapy
ISGPF: International Study Group for Pancreatic Fistula
LAMN: Low–grade appendiceal mucinous neoplasm
OS: Overall survival
PCI: Peritoneal Cancer Index
TA 60: Thoracoabdominal stapling device
