# Supplementary material for: Is the tail of the pancreas always tumor-infiltrated when macroscopically affected during cytoreductive surgery? A clinicopathological study and experience from a high-volume center
Source: World J Surg Oncol. 2025 Jul 24;23:300. doi: 10.1186/s12957-025-03954-4 (PMC12291515; doi:10.1186/s12957-025-03954-4)
Supplement: Supplementary file 1 — Supplementary Material 1 [file 12957_2025_3954_MOESM1_ESM.docx]

Is the tail of the pancreas always tumor-infiltrated when macroscopically affected during cytoreductive surgery? A clinicopathological study and experience from a high-volume center

Miklos Acs ^1^, Jozef Zustin ^2,3^, Niklas Bogovic ^1^, Pompiliu Piso ^4^, Sebastian Blaj ^4^

^1^ Department of Surgery, University Medical Center Regensburg, Regensburg, D-93053, Germany

^2^ Gerhard Domagk Institute of Pathology, University Medical Center Münster, Münster, D-48149, Germany

^3^ Institute of Pathology, University of Regensburg, Regensburg, D-93053, Germany

^4^ Department of General and Visceral Surgery, Hospital Barmherzige Brüder, Regensburg, D-93049, Germany

^*^ Corresponding author: [miklos.acs@ukr.de](mailto:miklos.acs@ukr.de)

**Table S1** Complete pre-, peri- and postoperative demographic and clinical characteristics of study participants. Patients were grouped based on whether the pancreas was infiltrated or not. The unit of frequency is the numbers of observations (percentage), while continuous data are presented as the mean ± standard deviation.

| **Clinical Characteristics** | **Total**  **(*N* = 31)** | **Without pancreatic tail infiltration**  **(*N* = 22)** | **With pancreatic tail infiltration**  **(*N* = 9)** | **Crude**  ***P* value** | **Adjusted *P* value** |
| --- | --- | --- | --- | --- | --- |
| Age (year) | 57.20 ± 13.34 | 57.84 ± 13.25 | 55.65 ± 14.22 | 0.6953 | 1.0000 |
| Sex |  |  |  | 1.0000 | 1.0000 |
| - Male | 14 (45.16%) | 10 (45.45%) | 4 (44.44%) |  |  |
| - Female | 17 (54.84%) | 12 (54.55%) | 5 (55.56%) |  |  |
| Primary tumor location |  |  |  | 0.6684 | 1.0000 |
| - Appendiceal neoplasms   -LAMN  -mucinous adenocarcinoma | 9 (29.03%)  8 (25.8%)  1 (3.2%) | 8 (36.36%)  8 (36.36%)  0 (0%) | 1 (11.11%)  0 (0%)  1 (11.11%) |  |  |
| - Colorectal cancer | 5 (16.13%) | 3 (13.64%) | 2 (22.22%) |  |  |
| - Gastric cancer | 7 (22.58%) | 4 (18.18%) | 3 (33.33%) |  |  |
| - Mesothelial cancer | 2 (6.45%) | 2 (9.09%) | 0 (0.00%) |  |  |
| - Ovarian cancer | 3 (9.68%) | 2 (9.09%) | 1 (11.11%) |  |  |
| - Peritoneal cancer | 5 (16.13%) | 3 (13.64%) | 2 (22.22%) |  |  |
| Pancreatic resection technique |  |  |  | 0.5642 | 1.0000 |
| - Open method | 6 (19.35%) | 4 (18.18%) | 2 (22.22%) |  |  |
| - endo-GIA | 18 (58.06%) | 14 (63.64%) | 4 (44.44%) |  |  |
| - TA 60 | 7 (22.58%) | 4 (18.18%) | 3 (33.33%) |  |  |
| Tumor grade ^1^ |  |  |  | 0.7538 | 1.0000 |
| - Highly differentiated (G1) | 8 (25.81%) | 6 (27.27%) | 2 (22.22%) |  |  |
| - Moderately differentiated (G2) | 9 (29.03%) | 5 (22.73%) | 4 (44.44%) |  |  |
| - Poorly differentiated (G3) | 7 (22.58%) | 5 (22.73%) | 2 (22.22%) |  |  |
| Mucinous tumor | 13 (41.94%) | 10 (45.45%) | 3 (33.33%) | 0.6860 | 1.0000 |
| Chemotherapy prior CRS + HIPEC | 25 (80.65%) | 17 (77.27%) | 8 (88.89%) | 0.6423 | 1.0000 |
| Radiotherapy prior CRS + HIPEC | 1 (3.23%) | 0 (0.00%) | 1 (11.11%) | 0.2692 | 1.0000 |
| Operated prior CRS + HIPEC | 25 (80.65%) | 18 (81.82%) | 7 (77.78%) | 1.0000 | 1.0000 |
| Peritoneal cancer index at exploration | 13.44 ± 7.66 | 15.28 ± 8.15 | 9.78 ± 5.19 | **0.0322** | **0.4186** |
| Duration of CRS + HIPEC (min) | 347.87 ± 115.36 | 352.91 ± 129.20 | 335.56 ± 76.47 | 0.9262 | 1.0000 |
| Duration of HIPEC |  |  |  | 0.4213 | 1.0000 |
| - 30-minutes | 9 (29.03%) | 6 (27.27%) | 3 (33.33%) |  |  |
| - 60-minutes | 17 (54.84%) | 11 (50.00%) | 6 (66.67%) |  |  |
| - 90-minutes | 5 (16.13%) | 5 (22.73%) | 0 (0.00%) |  |  |

**Table S1 (cont.)**

| **Clinical Characteristics** | **Total**  **(*N* = 31)** | **Without pancreatic infiltration**  **(*N* = 22)** | **With pancreatic infiltration**  **(*N* = 9)** | **Crude**  ***P* value** | **Adjusted *P* value** |
| --- | --- | --- | --- | --- | --- |
| CC score |  |  |  | 0.7446 | 1.0000 |
| - CC-0 | 23 (74.16%) | 17 (77.27%) | 6 (66.67%) |  |  |
| - CC-1 | 7 (22.58%) | 4 (18.18%) | 3 (33.33%) |  |  |
| - CC-2 | 1 (3.23%) | 1 (4.55%) | 0 (0.00%) |  |  |
| Surgical procedures |  |  |  |  |  |
| - Peritonectomy: parietal | 22 (70.97%) | 15 (68.18%) | 7 (77.78%) | 1.0000 | 1.0000 |
| - Peritonectomy: pelvis | 13 (41.94%) | 11 (50.00%) | 2 (22.22%) | 0.1296 | 0.7488 |
| - Peritonectomy: omental bursa | 16 (51.61%) | 15 (68.18%) | 1 (11.11%) | **0.0033** | **0.1716** |
| - Peritonectomy: right upper quadrant | 17 (54.84%) | 14 (63.64%) | 3 (33.33%) | 0.1059 | 0.6884 |
| - Peritonectomy: left upper quadrant | 24 (77.42%) | 17 (77.27%) | 7 (77.78%) | 0.6328 | 1.0000 |
| - Anastomosis: small bowel–small bowel | 9 (29.03%) | 6 (27.27%) | 3 (33.33%) | 1.0000 | 1.0000 |
| - Anastomosis: stomach–small bowel | 14 (45.16%) | 10 (45.45%) | 4 (44.44%) | 1.0000 | 1.0000 |
| - Anastomosis: small bowel–colon | 9 (29.03%) | 6 (27.27%) | 3 (33.33%) | 1.0000 | 1.0000 |
| - Anastomosis: colon–colon | 6 (19.35%) | 4 (18.18%) | 2 (22.22%) | 1.0000 | 1.0000 |
| - Anastomosis: colon–rectum | 7 (22.58%) | 7 (31.82%) | 0 (0.00%) | **0.0766** | 0.5690 |
| - Colostomy | 6 (19.35%) | 5 (22.73%) | 1 (11.11%) | 0.6328 | 1.0000 |
| - Ileostomy (protectively) | 2 (6.45%) | 2 (9.09%) | 0 (0.00%) | 1.0000 | 1.0000 |
| - Stomach resection | 16 (51.61%) | 12 (54.55%) | 4 (44.44%) | 1.0000 | 1.0000 |
| - Small bowel resection | 8 (25.81%) | 3 (13.64%) | 5 (55.56%) | **0.0159** | **0.4134** |
| - Colon resection | 18 (58.06%) | 12 (54.55%) | 6 (66.67%) | 0.6961 | 1.0000 |
| - Rectosigmoid resection | 8 (25.81%) | 8 (36.36%) | 0 (0.00%) | **0.0705** | 0.5690 |
| - Splenectomy | 25 (80.65%) | 18 (81.82%) | 7 (77.78%) | 1.0000 | 1.0000 |
| - Cholecystectomy | 22 (70.97%) | 13 (59.09%) | 9 (100%) | **0.0315** | **0.4186** |
| - Liver metastectomy | 10 (32.26%) | 7 (31.82%) | 3 (33.33%) | 1.0000 | 1.0000 |
| - Greater omentectomy | 20 (64.52%) | 16 (72.73%) | 4 (44.44%) | 0.2175 | 1.0000 |
| - Lesser omentectomy | 16 (51.61%) | 12 (54.55%) | 4 (44.44%) | 0.7043 | 1.0000 |
| - Hysterectomy | 4 (12.90%) | 3 (13.64%) | 1 (11.11%) | 1.0000 | 1.0000 |
| - Ovarectomie | 3 (9.68%) | 3 (13.64%) | 0 (0.00%) | 0.5448 | 1.0000 |
| - Resection of other organs | 11 (35.48%) | 9 (40.91%) | 2 (22.22%) | 0.6722 | 1.0000 |
| Length of hospital stay (day) | 24.55 ± 13.82 | 24.23 ± 15.23 | 25.33 ± 10.28 | 0.4833 | 1.0000 |
| Length of intensive care unit stay (day) | 7.23 ± 6.47 | 7.14 ± 5.76 | 7.44 ± 8.28 | 0.2388 | 1.0000 |

**Table S1 (cont.)**

| **Clinical Characteristics** | **Total**  **(*N* = 31)** | **Without pancreatic infiltration**  **(*N* = 22)** | **With pancreatic infiltration**  **(*N* = 9)** | **Crude**  ***P* value** | **Adjusted *P* value** |
| --- | --- | --- | --- | --- | --- |
| Complication grade (Clavien–Dindo) |  |  |  | 0.6819 | 1.0000 |
| - I | 10 (32.26%) | 7 (31.82%) | 3 (33.33%) |  |  |
| - II | 3 (9.68%) | 3 (13.64%) | 0 (0.00%) |  |  |
| - IIIA | 10 (32.26%) | 6 (27.27%) | 4 (44.44%) |  |  |
| - IIIB | 6 (19.35%) | 5 (22.73%) | 1 (11.11%) |  |  |
| - IV | 2 (6.45%) | 1 (4.55%) | 1 (11.11%) |  |  |
| Postoperative complications |  |  |  |  |  |
| - Pancreatic fistula | 8 (25.81%) | 6 (27.27%) | 2 (22.22%) | 1.0000 | 1.0000 |
| - Surgical site infection | 2 (6.45% | 2 (9.09%) | 0 (0.00%) | 1.0000 | 1.0000 |
| - Pleural effusion | 6 (19.35%) | 2 (9.09%) | 4 (44.44%) | **0.0434** | **0.4514** |
| - Thrombosis | 3 (9.68%) | 1 (4.55%) | 2 (22.22%) | 0.1949 | 1.0000 |
| - Pneumonia | 5 (16.13%) | 4 (18.18%) | 1 (11.11%) | 1.0000 | 1.0000 |
| - Urinary tract infection | 2 (6.45%) | 2 (9.09%) | 0 (0.00%) | 1.0000 | 1.0000 |
| - Anastomotic leak | 1 (3.23%) | 1 (4.55%) | 0 (0.00%) | 1.0000 | 1.0000 |
| - Acute Respiratory Distress Syndrome | 1 (3.23%) | 0 (0.00%) | 1 (11.11%) | 0.2903 | 1.0000 |
| - Sepsis | 1 (3.23%) | 1 (4.55%) | 0 (0.00%) | 1.0000 | 1.0000 |
| - Pulmonary embolism | 3 (9.68%) | 3 (13.64%) | 0 (0.00%) | 0.5375 | 1.0000 |
| - Reoperation | 8 (25.81%) | 6 (27.27%) | 2 (22.22%) | 1.0000 | 1.0000 |
| Median survival time [months (95% CI)] | 21.22  (17.15 – not reached) | 23.85  (17.15 – not reached) | 20.78  (8.57 – not reached) | 0.5020 | 1.0000 |

CC: Sugarbaker’s completeness of cytoreduction score; CRS: cytoreductive surgery; LAMN: low grade appendiceal mucinous neoplasm; endo-GIA: endo gastrointestinal anastomosis stapling device; HIPEC: Hyperthermic Intraperitoneal Chemotherapy; TA 60: thoracoabdominal stapling device.

^1^ Not all patients’ data contained information about this parameter.
